# Children’s Ages, Reasons, and Experiences for First Dental Visit and Barriers of a Late Visit: A Cross-Sectional Community-Based Study in Saudi Arabia

**DOI:** 10.7759/cureus.60942

**Published:** 2024-05-23

**Authors:** Shahad S Alkhuwaiter, Manahil Almutairi, Norah Alfuraih, Sanaa N Al-Haj Ali

**Affiliations:** 1 Department of Orthodontic and Pediatric Dentistry, College of Dentistry, Qassim University, Qassim, SAU; 2 General Dentistry, College of Dentistry, Qassim University, Qassim, SAU

**Keywords:** barriers, reason, age, parents, children, first visit, dental visit

## Abstract

Objective: This study aimed to determine the age and reason of pediatric patients at the first visit and the barriers to a late visit.

Methodology: A study was conducted among guardians of children attending the Outpatient Pediatric Dentistry Department. Children who visit the dentist for the first time were included in the study. A self-administered questionnaire was used and the behavior of children was evaluated by using Frankl's scale. Descriptive statistics was used to explore the general data.

Results: A total of 211 children had their first dental visit. The majority (n = 112, 53.1%) visited the dentist for the first time at the age of three to six years. Reasons for the first dental visit for most children were dental caries (n = 118, 32.8%) followed by dental pain (n = 114, 31.7%). More than half of the parents (n = 160, 75.8%) reported that the overall experience of the first visit was very good. The highest reported barriers to a late dental visit were that the child's not complaining of dental problems (n = 60, 20.5%) and dental fear and anxiety (n = 58, 19.8%).

Conclusions: Most of the children in this study visited the dentist for the first time beyond the internationally recommended age and the reason behind this visit is to relieve a specific chief complaint. Moreover, the barriers contributing to the postponement of a child's first dental visit vary in this study. The child not complaining of any dental problems and dental fear and anxiety were the highest reported barriers.

## Introduction

The American Academy of Pediatric Dentistry recommends that children visit a dentist by their first birthday or within six months after their first tooth emerges. This early visit helps establish a dental home for the child and allows the dentist to monitor oral health and provide guidance on oral care practices for parents [1,2].

The first visit of children to a pediatric dentist is an important milestone in their oral health journey and it is crucial for several reasons. First is early introduction and detection, the first visit allows children to become familiar with the dental environment in a friendly and supportive manner, and at the same time it allows the dentist to identify any potential oral health issues early on, such as dental caries, or any developmental abnormalities. Second, a dental home helps establish a relationship between the child, parent, and dentist, creating a comfortable and familiar environment for future visits [3]. Guidance on proper dental care measures for oral/dental problems prevention and primary intervention if necessary such as fluoride varnish, dental sealants, or orthodontic interventions to maintain optimal oral health [4]. Parent education is another crucial factor. During the first appointment, parents can get advice on age-related guidance on proper oral hygiene practices, diet, fluoride use, and other areas of their children's dental care. In addition, the dentist also can respond to any queries or concerns parents may have and offer individualized recommendations for their child's oral health [5]. Overall, the first dental visit sets the foundation for lifelong healthy oral hygiene practices and ensures that any issues are addressed promptly, improving their quality of life in the future [6].

Previous studies reported factors for a child's dental visit being delayed, including lack of awareness: parents may not be aware of the importance of early dental visits for children [7]. Financial constraints: cost can be a barrier to accessing dental care for some families, especially if they do not have insurance coverage. Fear or anxiety: children or parents may feel anxious or fearful about visiting the dentist, which can lead to postponing appointments [8]. Busy parents may find it challenging to prioritize dental appointments due to conflicting priorities or active schedules. Finally, there are geographic constraints. Some families may reside in places where access to dental care is restricted, which makes it difficult to make timely appointments [9].

Collecting data regarding the age at which the child visited the dentist, the reasons for the visit, and addressing the barriers of a late visit are crucial for establishing a guideline and recommendations for the parents, with emphasis on dental education, community outreach, financial assistance programs, and dental public health initiatives that can help improve access to timely dental care for children.

Thus, this study aimed to determine the age, reason, behavior of pediatric patients, and the provided dental treatment during their first dental visit; assess the parents’ experiences after receiving the first dental visit of their children; and discuss the barriers of a late visit to the dentist at Al-Qassim region of Saudi Arabia.

## Materials and methods

A cross-sectional questionnaire-based study was conducted among guardians of children attending the Outpatient Pediatric Dentistry Department at Qassim University College of Dentistry, Qassim. Ethical approval was taken from the Scientific Research Committee, Qassim University, before conducting the study (IRB #23-27-09). Informed consent was obtained from the parents. A convenient sample of 211 participants was included in the study. All the parents (mother or father)/guardians willing to participate in the study and whose children visit the dentist for the first time were included in the study. Those parents/guardians whose children suffered from any mental/physical disability or syndromes were excluded from the study. A self-administered 11-item questionnaire was developed and validated by Alshahrani et al. [10]. It was modified to align with the objectives of the study, then it was translated into the local Arabic language using World Health Organization methodology for ease of understanding of the participants. After that, the questionnaire was translated back into English. The bilingual authors confirm the similarity between the original English questionnaire and the translated Arabic version. The questionnaire was pilot-tested on 15 participants to examine the validity and analysis of the question flow and whether it captures the information it intends to measure by offering feedback about their experiences of filling it out.

The questionnaire involved questions related to age at the first dental visit of a child, the medical health of the child, reasons for the first dental visit, selection of place of treatment, children's behavior during the first dental appointment, the general experience and the care provided at the first appointment, reasons to attend/miss the recall dental visits, and also, main barriers of a late visit.

The behavior of children at the first visit was evaluated by using Frankl's scale, which ranges from definitely negative to definitely positive. The participants were assured that their responses would not be disclosed to maintain confidentiality. After the participants had been surveyed for half an hour, the questionnaires were gathered. Hence, data from the duly filled (211) questionnaires were analyzed statistically using IBM SPSS software. Descriptive statistics (frequencies and percentages) were used to explore the general data.

## Results

The data was entered and analyzed using IBM SPSS software. Descriptive statistics (frequencies and percentages) were used to explore the general data. A total of 211 children had their first dental visit. The majority (n = 112, 53.1%) of children in this study visited the dentist for the first time at the age of three to six years while the lowest percentage (n = 1, 0.5%) visited the dentist for the first time at the recommended age zero to one year. Dental caries (n = 118, 32.8%) and pain (n = 114, 31.7%) were the dominant reasons for the first dental visit, meanwhile, the least reasons were referred by a dentist (n = 3, 0.8%) or medical personnel (n = 3, 0.8%). The quality of provided treatment is the main reason for selecting the place of treatment (n = 87, 41.2%), and at this university dental hospital, about 65% who provided the treatment for the children were dental interns (Table 1).

**Table 1:** Age and reasons for the first dental visit

Age	N (%)
0-1 year	1 (0.50)
>1-3 years	13 (6.20)
>3-6 years	112 (53.10)
>6-9 years	57 (27)
>9 years	28 (13.30)
Reason	N (%)
Pain	114 (31.7%)
Dental caries	118 (32.8%)
Abscess/swelling	30 (8.3)
Routine dental check-up	22 (6.1)
Retained deciduous tooth	21 (5.8)
Fluoride application	15 (4.2)
Tooth discoloration/stains	15 (4.2)
Trauma	9 (2.5)
Malocclusion	10 (2.8)
Referred by dentist	3 (0.8)
Referred by medical personnel	3 (0.8)

The treatment provided to more than half of the children (n = 126, 59.7%) at the first dental visit was relief of the chief complaint, and the least was giving dental advice only (n = 4, 0.7%). Most of the parents (n = 188, 89.1%) reported that their child's problem was treated during the first visit, so 59.7% will keep the next appointment for their children. On the other hand, 10.9% of the parents were not sure about keeping their next appointment, and the main reason was busy at work (41.7%) (Table 2).

**Table 2:** Frequency distribution of different aspects at the first dental visit

Treatment provided at the first dental visit	N (%)
The chief complaint of the child was treated	126 (59.7)
Only oral examination/x-ray done	74 (35.1)
Only medication prescribed	2 (0.9)
Only dental advice given	4 (1.9)
Referred to the pediatric dentist for treatment	5 (2.4)
Reason for selecting the place of treatment	N (%)
Quality of provided treatment	87 (41.2%)
Advice from friends/relatives	70 (33.2%)
Previous personal experience	44 (20.9)
Near distance from home	5 (2.4)
Other reason	5 (2.4)
Treatment provider to the child	N (%)
General practitioner	12 (5.7)
Pediatric dentist	5 (2.4)
Intern	129 (61.1)
Dental student	61 (28.9)
I don’t know	4 (1.9)

About half of the children in this study behaved definitely positively at their first dental visits, and only 8.1% showed a definitely negative behavior. Overall, 75.8% (n = 160) of the parents perceived that the experience of the first visit of their children was very good (Table 3).

**Table 3:** Child’s behavior and the experience of the first visit

Frankl Behavioral Rating Scale	N (%)
Definitely negative	17 (8.1)
Negative	28 (13.3)
Positive	60 (28.4)
Definitely positive	106 (50.2)
Overall experience of the first dental visit	N (%)
Very good	160 (75.8)
Good	36 (17.1)
Satisfactory	15 (7.1)
Will keep the next visit?	N (%)
Yes	188 (89.1)
No	23 (10.9)
Reason to keep next visit	N (%)
The treatment provided relieved my child’s problem	90 (28.6)
Good behavior of dental professionals	77 (24.4)
Importance of dental treatment	77 (24.4)
For completing my child’s treatment plan	71 (22.5)
Reason to miss next visit	N (%)
The treatment provided did not relieve my child’s problem	6 (25)
Further dental treatment not required	3 (12.5)
Busy at work	10 (41.7)
Other reason	5 (20.8)

Evaluating the barriers of a late dental visit determined that the highest three reported barriers by the parents were their child not complaining of any dental problems (n = 60, 20.5%), dental fear and anxiety (n = 58, 19.8%), and (n = 46, 15.7%) belief that primary teeth are not important like permanent teeth (Table 4).

**Table 4:** Barriers of a late dental visit

Barrier	N (%)
Dental fear and anxiety	58 (19.8)
Primary teeth are not as important as permanent teeth	46 (15.7)
No real need to go to the dentist	14 (4.8)
My child is not complaining of dental problems	60 (20.5)
I live in a remote area	9 (3.1)
Transportation barriers	35 (11.9)
Difficulty of getting a dental appointment	24 (8.2)
Unsuitable appointment times	23 (7.8)
Long wait times during my child's treatment	15 (5.1)
Financial barriers	7 (2.4)
Other barrier	2 (0.7)

## Discussion

Children's first dental visit is a pivotal milestone in their healthcare journey, and the timing of this crucial appointment often varies between the parents. Understanding the reasons behind first visit delay among children is important for improving overall oral health. In this study, most of the children visited the dentist for the first time beyond the internationally recommended age. The reason behind this visit is to relieve a specific chief complaint (dental caries followed by pain). Moreover, the barriers contributing to the postponement of a child's first dental visit vary in this study. Children’s parents believed that if their child had no dental complaint, no need to visit the dentist. This reflects that they are unaware of the importance of the first dental visit at an early age. Our investigation aimed to shed light on the nuanced interplay between parental perceptions, cultural beliefs, and their impact on the timing of this fundamental dental encounter. Through a comprehensive analysis of these factors, we seek to provide insights that can guide targeted interventions and educational strategies to promote early dental care, ultimately fostering improved oral health practices among children. This discussion section delves into the findings, implications, limitations, and potential avenues for further research, underscoring the significance of addressing age-related reasons for delayed first dental visits in pediatric dentistry.

Age at first dental visit

An early dental visit during a child's formative years is essential to ensure their timely access to professional dental care and preventive services [2]. As per the guidelines from the American Academy of Pediatric Dentistry, it is recommended to schedule the initial clinical examination when the first primary tooth emerges, that is, ideally before the child reaches 12 months of age. A child's first dental visit at the age of one year old sets the stage for a lifetime of learning about preventive dental care, helping to pave the way for good oral health as they grow up [4]. This study shows that most of the children visited the dentist for the first time when they were between the ages of three and six years, and a small number of children visited when they were younger than three years old. Previous reports in multiple regions of KSA such as Riyadh and Abha found that most children typically have their first dental visit between the ages of three and five years old [4,7]. Similar studies also reported that in Saudi society, parents or guardians expressed the belief that a dental visit before a child reaches one year of age was inappropriate [3,11]. Instead, most preferred scheduling the first dental visit between the ages of three and six years. This inclination was based on worries about the child's likelihood of displaying uncooperative behavior or non-compliance during that early period.

Reasons for first dental visit

The primary reasons identified for the late initial dental visits across different studies were predominantly pain and dental caries. This study's outcomes mirrored previous research, indicating that decayed teeth followed by pain were the most prevalent reasons. This consistency aligns with earlier studies conducted in Saudi Arabia, where parents or guardians typically introduced their children to dentists between the ages of three and six years [4,7]. This trend originates from a misconception among parents, presuming that their children do not need to see dentists or pediatric dentists unless a problem arises, particularly before the child turns one year old. 

In this study, there were low percentages of parents who sought early dental care for their children for preventive purposes like check-ups or fluoride applications. These findings indicate a noticeable lack of dental awareness and understanding regarding the importance of primary teeth among the parents involved in the study. Baghdadi observed similar attitudes among parents in Saudi society, revealing an undervaluation of the role that primary teeth, specifically, play in the overall health and well-being of their children [12].

Children’s behavior at first dental visit

To assess the child's behavior, Frankl's scale was utilized, known for its reputation as the most dependable scale for evaluating children's conduct in a clinical environment [6]. About 50.2% of the children were found to have defiantly positive behavior during their first dental visit. The results aligned with those of prior studies conducted in multiple regions of Saudi Arabia, where the majority of participants were documented to have displayed positive behavior during their first dental visit [3,4,7]. The positive behavior when considering their older ages, the predictability of this outcome was not surprising. This aligns with the straightforward nature of explaining preventive measures. Furthermore, an initial dental appointment commonly involves an introduction to the dental setting, prophylaxis, guidance on oral hygiene, and the utilization of X-rays, while the negative behavior exhibited by a child during their first dental visit when it is delayed could arise from various factors [13]. One reason could be if pain or discomfort has developed due to the delayed visit. Overall, delayed visits might contribute to heightened anxiety or negative perceptions about dental care, influencing the child's behavior during the first visit.

During dental procedures, children's anxiety levels often rise, but fostering a positive attitude among both children and parents toward dentistry and using diverse behavior management techniques such as positive reinforcement or voice control can help ease these anxieties [14]. Parents/guardians have a significant influence over children’s behavior and their feelings about dental treatment, which significantly impacts children's oral health. Throughout childhood, parents can mold their children's behavior by guiding and discouraging specific habits [15].

There are factors influencing the choice of treatment location and the likelihood of parents keeping the next dental appointment for their children. A substantial proportion of participants (41.2%) identified the quality of provided treatment as the primary reason for selecting a specific treatment place. Concurrently, the majority of the treatment provided by dental interns (65.3%) indicated this may stem from various considerations, including accessibility, cost-effectiveness, and a positive perception of the skills and capabilities of dental interns. Encouragingly, almost 60% of participants expressed their intention to keep the next appointment for their children, with a dominant reason being the perceived efficacy of the treatment in alleviating their child's dental chief complaint (89.1%). However, 10.9% expressed uncertainty about keeping the next appointment, primarily attributing it to being overly occupied at work (41.7%). These findings underscore the importance of perceived treatment quality and provider qualifications in influencing treatment location preferences while highlighting the potential impact of work commitments on the commitment to future dental appointments. Addressing these factors could contribute to enhancing overall dental care experiences for children and their parents.

Barriers

Several barriers were identified concerning the first dental visit, with the most prevalent being the perception that a child not complaining about dental problems, reported by 20.5% of participants. Additionally, dental fear and anxiety were cited as significant barriers by 19.8% of respondents, while a belief that primary teeth are not as crucial as permanent teeth constituted a barrier for 15.7% of participants. Conversely, unspecified other barriers were the least reported, accounting for only 0.7%. These findings underscore a range of factors contributing to the delay in initiating dental visits for children, reflecting both attitudinal barriers, such as minimizing the importance of primary teeth, and emotional barriers, such as dental fear and anxiety. Addressing these barriers is essential for promoting timely and preventive dental care for children.

This study has some limitations that may have affected the overall results. The data collection was only at the Dental University Hospital and the data collection period was limited, which could have been longer with a larger sample size. Future studies including different primary health care centers and hospitals in the AlQassim region of Saudi Arabia are strongly recommended. Also, there is an increased need for prevention, promotion, and education programs in Saudi communities to increase awareness about the importance of visiting the dentist at the recommended age and taking oral health care of children at the earliest stage of their life. Furthermore, all dental professionals, pediatricians, and primary healthcare providers have to work together to create awareness regarding regular dental examinations and the importance of early referral to dental clinics. Another limitation of this study is the convenient sampling technique.

## Conclusions

Most of the parents in this study are unaware of the appropriate age of their children's first dental visit that is recommended by the major pediatric dental academies. Parents brought their children to the dentist after their children experienced dental caries or suffered from pain. There was no complaint of any dental problems by the children and dental fear and anxiety are the two major barriers of a late visit to the dentist.
